# Authorship identification using ensemble learning

**DOI:** 10.1038/s41598-022-13690-4

**Published:** 2022-06-09

**Authors:** Ahmed Abbasi, Abdul Rehman Javed, Farkhund Iqbal, Zunera Jalil, Thippa Reddy Gadekallu, Natalia Kryvinska

**Affiliations:** 1 Department of Creative Technologies, PAF Complex, E-9, Air University, Islamabad, Pakistan; 2 Department of Cyber Security, PAF Complex, E-9, Air University, Islamabad, Pakistan; 3 College of Technological Innovation, Zayed University, Abu Dhabi, UAE; 4 School of Information Technology and Engineering, Vellore Institute of Technology, Vellore, India; 5 Information Systems Department, Faculty of Management, Comenius University in Bratislava, Odbojárov 10, 82005 Bratislava 25, Slovakia

**Keywords:** Computer science, Information technology

## Abstract

With time, textual data is proliferating, primarily through the publications of articles. With this rapid increase in textual data, anonymous content is also increasing. Researchers are searching for alternative strategies to identify the author of an unknown text. There is a need to develop a system to identify the actual author of unknown texts based on a given set of writing samples. This study presents a novel approach based on ensemble learning, *DistilBERT*, and conventional machine learning techniques for authorship identification. The proposed approach extracts the valuable characteristics of the author using a count vectorizer and bi-gram Term frequency-inverse document frequency (TF-IDF). An extensive and detailed dataset, “All the news” is used in this study for experimentation. The dataset is divided into three subsets (article1, article2, and article3). We limit the scope of the dataset and selected ten authors in the first scope and 20 authors in the second scope for experimentation. The experimental results of proposed ensemble learning and *DistilBERT* provide better performance for all the three subsets of the “All the news” dataset. In the first scope, the experimental results prove that the proposed ensemble learning approach from 10 authors provides a better accuracy gain of 3.14% and from *DistilBERT* 2.44% from the article1 dataset. Similarly, in the second scope from 20 authors, the proposed ensemble learning approach provides a better accuracy gain of 5.25% and from *DistilBERT* 7.17% from the article1 dataset, which is better than previous state-of-the-art studies.

## Introduction

**Figure 1:**
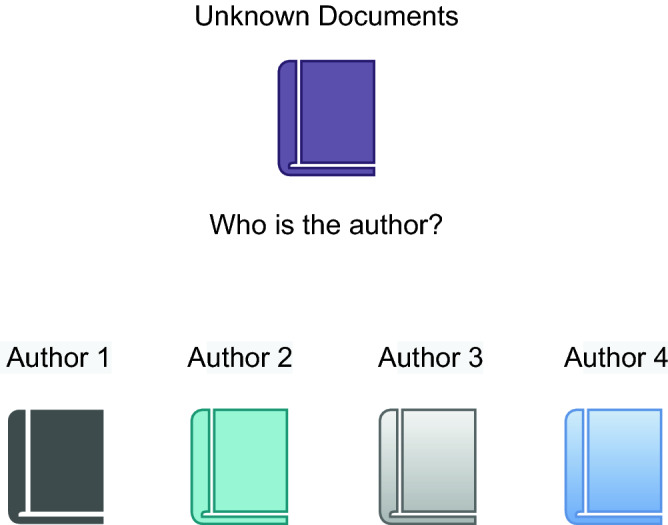
Authorship identification process.

Recently, authorship identification has gained significant attention in the research community^1^. The identification of authorship of handwritten textual documents is an ancient way^2^. Now, the massive quantity of textual content is available in a digital form and stored in various unstructured formats^3, 4^. Text mining plays an essential role in author identification. Extracting meaningful information from unstructured or semi-structured formats is also a challenging task^5^. Text mining is widely used to analyze a large amount of unstructured data and extract meaningful insights^6^. Text mining aims to extract meaningful information from the text data, which is present in unstructured or semi-structured formats. Text mining uses machine learning (ML), and natural language processing (NLP) techniques to create text analysis models to extract or classify specific information based on training data^7, 8^. Figure 1 shows the authorship identification process. Author identification is the task of identifying the feasible author of unknown documents from multiple candidate authors. The authorship identification is usually taken into consideration by a text classification task. It starts with pre-processing a dataset, then features extraction and selection, converting the textual data into a feature vector. Feature engineering is the essential step in machine learning (ML) used to predict the model. Recently, authorship identification applications have been developed in numerous fields like cybercriminals law^9^, opinion analysis detection system^10^. AI is also a part of cryptography detection, signature detection, and intrusion detection. The main and challenging task of authorship identification is to extract the most important features representing the author’s writing style. Being able to extract the most important features might enable accurate authorship identification. Many researchers have worked on this domain and suggested several solutions^11, 12^. The most important characteristics like lexical^13^, syntactic^14^, content specific^15^ and stylometric features^16^ are used for authorship identification. Furthermore, there are many words embedding feature extraction techniques that are used in NLP text classification and text mining tasks^17–19^. This technique extracts the relevant characteristics from the text data. It also provides a word vectors database that is mainly used to enable better classification performance of ML algorithms^20^.

Many traditional approaches have been used for authorship identification, which presents the analyses by mining text and authorship classification using NLP techniques. In this study, we use the “All the news” dataset to identify the authors of the news article. Text mining, identification and classification of authors, and extraction of authors’ writing styles from various techniques are analyzed using the proposed methodology.

This study proposed an authorship identification system combined with two feature extraction techniques that extract the information related to each author’s writing style. The proposed approach depends on mainly two steps. The first step is selecting maximum suitable characteristics to explain the multiple authors writing styles from unstructured text documents. On the other hand, the second step is selecting multiple algorithms (RF, XGB, MLP, LR, Ensemble, and *DistilBERT*) to identify and classify the authors that belong to the actual text. The discussion and the comparison of authorship identification and classification mechanisms is one of the essential contributions of this study. The proposed bi-gram TF-IDF and Count vectorizer feature engineering technique ultimately enhance the authorship identification and classification performance than classical approaches presented in the past presented in section "Literature review". The news articles were collected using Beautifulsoup, which extracts the data in a hierarchical and more readable manner and then chopped it up into three parts (article1, article2, and article3). We used various algorithmic models to train and validate the “All the news” dataset to identify and classify authors. The main contributions of this study are:

- We propose an implicit ensemble learning and Multi-Depth technique comprising multiple classifiers to participate in the voting-based decision for authorship identification and classification. The voting process depends upon a threshold; a classifier assigns a vote to the input data when the confidence level has passed.
- Propose a framework to extract authors-related concise information from textual data using Count vectorizer and term frequency-inverse document frequency (TFIDF) that automatically learn features without human interference.
- Proposed framework outperforms other state-of-the-art Machine learning and natural language processing methods that use various feature extraction techniques. The proposed ensemble learning technique and Transformation-based model multi-depth *DistilBERT* performed well on news articles datasets among earlier baseline methods.

The rest of this paper is organized as follows. A detailed illustration of the past state-of-art work is provided in section "Literature review". A brief explanation of the dataset is presented in section "Dataset selection". The proposed ensemble and Multi-Depth *DistilBERT* approach is explained in section “Proposed approach”. The detailed explanation of experiments and results are presented in section “Experimental analysis and results”, in the end, we provide a conclusion in section “Conclusion” and limitations and future work of this study in section “Limitation and future work”.

## Literature review

This section presents state-of-art research on authorship analysis based on the author’s writing style features, analytical strategies, more than one language problem, and different associated parameters. In the end, we suggest a taxonomy for authorship analysis research.Please confirm the section headings are correctly identified It is correct.

### Authorship analysis

Authorship identification of handwritten documents began as early as the late 19th century, but now the vast volume of textual data is digital. However, it was only recently that researchers started investigating authorship identification of digital textual content stored in unstructured formats^21, 22^. The authorship analysis analyzes the characteristics and concise information related to the author’s writing style to conclude its authorship^14^. Authorship analysis is based on stylometry, the branch of linguistics; eventually, it has improved with more advanced methods and techniques by using ML and NLP. Authors in^23^ proposed an approach that tried to resolve the problem of the Federalist Papers. Authors in^24^ worked on a study related to software forensics. They worked on four essential authorship analysis regions: authorship detection, authorship characterization, similarity identification, and authorship discrimination.

### Approaches to authorship identification

This study shows that there are mainly four approaches that are most suitable for authorship identification^25^. One of them is keystroke biometrics. It is based on the usage of software applications to produce features based on the manner and rhythm in which an author types characters on a keyboard or keypad. The primary and popular approach used in authorship identification is the stylometry-based approach. It has been used in several approaches over the last few years. It uses the author’s writing style and extracts the essential attributes of the document^26^. Most importantly, there are four primary types of stylometric functions precisely. Lexical features, content-specific features, syntactic capabilities, and structural features^27^.

### Drawbacks of the traditional approaches

As stated by Zhou and Wang, there were above 1000 various features used for authorship analysis in the past research. Still, no work shows the most effective and useful features, and no research has proven that various sets of features are more suitable for different application settings^28^. The experimental results heavily rely on selected features and chosen classifiers. Therefore, there is a need to improve the feature extraction technique so that only the most relevant features are selected. The first approach, keystroke biometrics for authorship identification, is described above. It has been applied in various studies and performed very well. The main advantage of this technique is that it can be installed in computer software programs and used by authors. Still, it is not easy to manage while organizing remote examinations.

On the other hand, the second linguistic approach is too sophisticated and unsuitable and straightforward for our modern world. In contrast to the popularity and validity of the stylometry-based approach, we noticed some limitations and drawbacks. The first and primary task is the selection of the features which are used for authorship identification. According to de vel, every single author has specific characteristic features that lead to better performance^29^. However, it is still a challenging task to define a universal feature extraction technique that can be used everywhere because currently, features are limited to specific applications^30^. The third approach is authorship identification. It has been popular in the recent past due to data available in digital form. Still, there are some issues because the data is limited to the particular author. The data is language-dependent which is the most challenging task of this approach. This issue was highlighted by Zheng et al., which attempt to identify authorship attribution to online messages in English and Chinese languages^14^.

### Language modelling for authorship identification

Language modeling is highly used for several NLP-based speech recognition and email classification applications. In language modeling, the model will be given a sequence of words, and it has to give the probability of what should be the next word. Over time, the usage of language models has additionally located its manner into the authorship analysis. The authors used English, Chinese, and Greek language data and applied character-level language modeling^30^. This novel technique has attained good results using all the languages. This technique proves that this approach is independent of the language in use. The fundamental concept behind the usage of this method is to train a separate language model for every single author. Therefore, to identify textual data, whether it belongs to the predicted author or not, needs to feed the data to a specific model trained on a particular writer’s writing style. The model will generate the probability of the textual data, whether it belongs to the author or not.

We use unseen data to identify how likely that author wrote it during the prediction phase. It would generate a high prediction value if that author wrote the textual data. This is the working process of language models for authorship attribution. If you deal with more than one language model, each model will be trained on a specific author’s writing. There will be no change in the model’s architecture; the model will be the same; change the training dataset to train the model to identify individual authors. These approaches also have some disadvantages, primarily related to stylometry-based approaches that have some problems during the selection of specific features and also some language dependencies.

The proposed approach identifies the most suitable features using the count vectorizer and bi-gram TF-IDF related to all the earlier work. This approach also attained bench-marked results using an ensemble learning approach and an NLP-based language model to identify authors. We compare the proposed approach with the baseline approach, which shows that we outperform the baseline approach with better performance.

## Dataset selection

**Figure 2:**
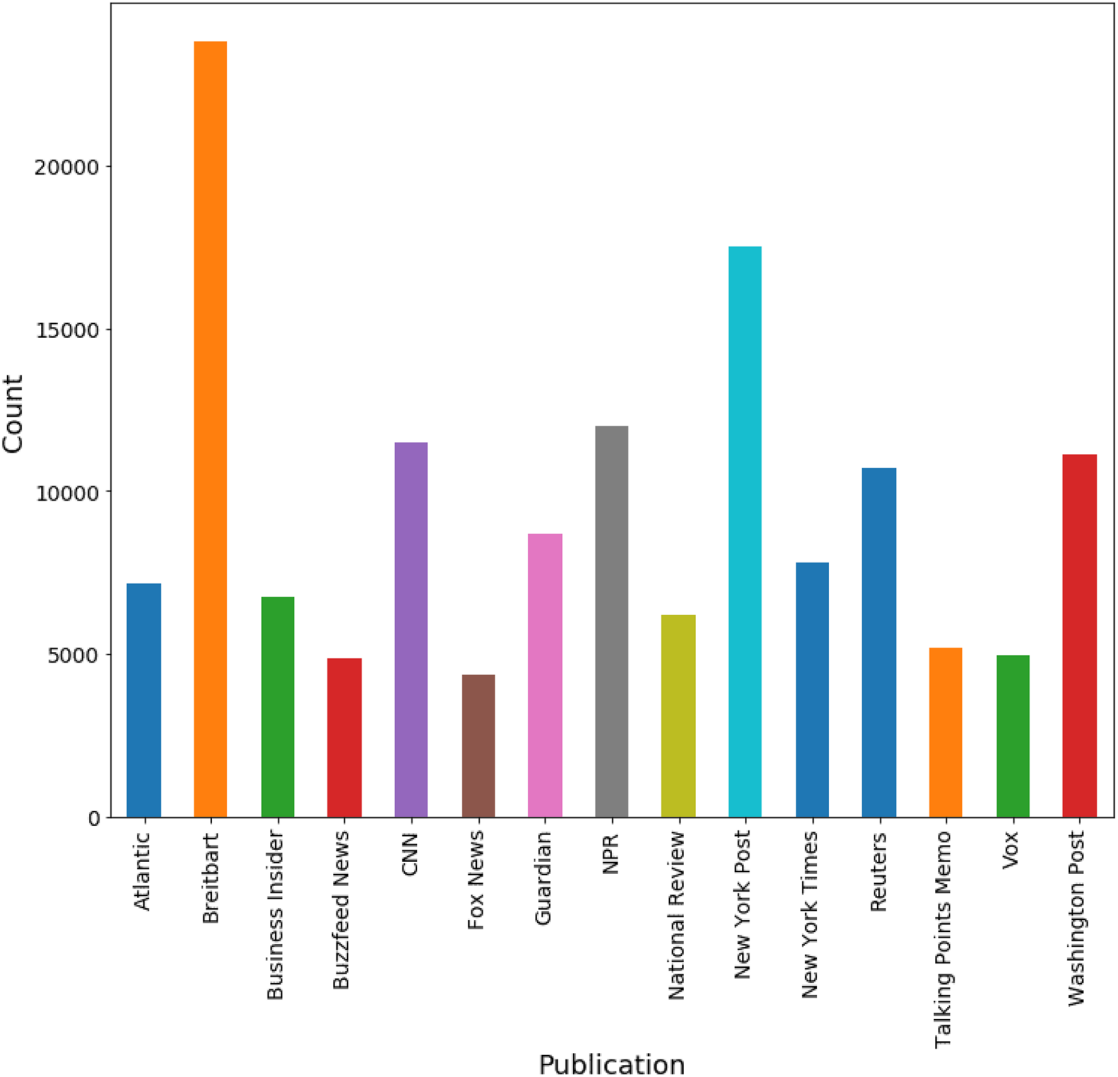
Total number of publications with article count.

With time, the textual data is growing exponentially via published articles. Identifying text that belongs to which author is such a challenging task, so we used the author identification process to identify the deserving author significantly. In this study, we used the “all the news” dataset available on kaggle^31^. This dataset contains various publications, and the number of news articles per publication is shown in Fig. 2. Breitbart and New York Post are the top 2 publications containing 143,000 news articles. The dataset contains articles from 2000 to 2017, but most of the published articles from 2016 and 2017 cover various topics. This dataset consists of 9 attributes (ID, Title, Publication, Author, Date, Year, Month, URL, and Content) and 143,000 news articles collected from 15 different sources. To extract the features, we used the ’Content’ attribute from the dataset that contains the actual text of the news articles, and for prediction, we used the ’author’ attribute as a target column for model prediction.

The author in^31^ divided the dataset into three subsets: articles1, articles2, and articles3. The subsets (articles1, articles2, and articles3) all have the same number of features (9 features): ID, which is the database ID and is an integer type, title, which is the title of the article and is a string type, publication, which contains the names of the publications, the fourth feature contains the names of the authors, then the date of publication, year of publication, the month of publication, the URL of that article, and finally feature named ’Content’ contains the textual content of the article.

.

**Figure 3:**
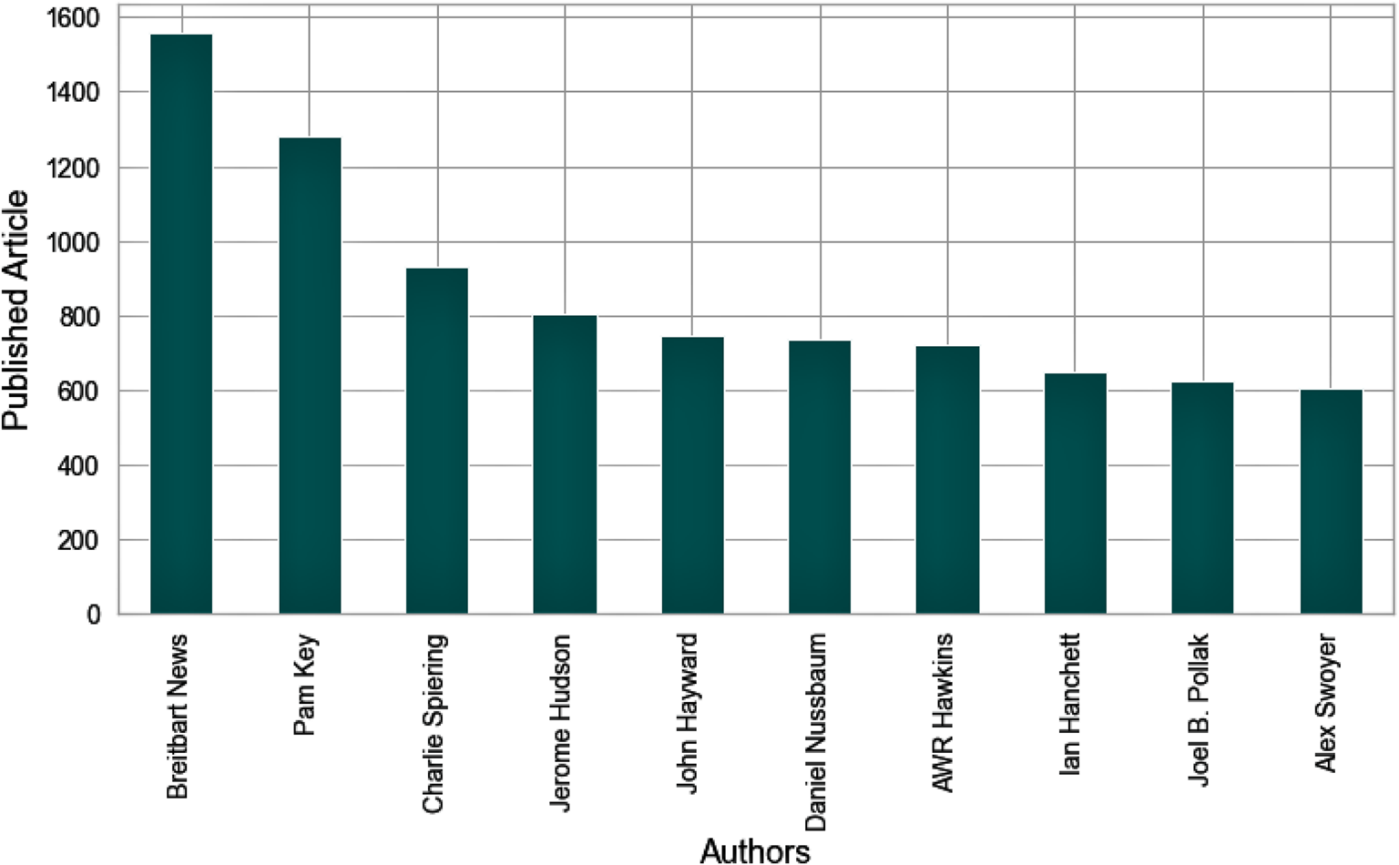
Top 10 authors in article1 dataset.

**Article1:** The article1 dataset consists of 9 attributes and 50,000 articles from 3603 authors fetched from 5 publications. The average number of words in the article1 dataset is 668, and the most comprehensive article has 26,297 words. The *Breitbart Author* published the most articles in the Article1 dataset. Figure 3 presents the top 10 authors that published the most articles in article1 dataset publications.

**Figure 4:**
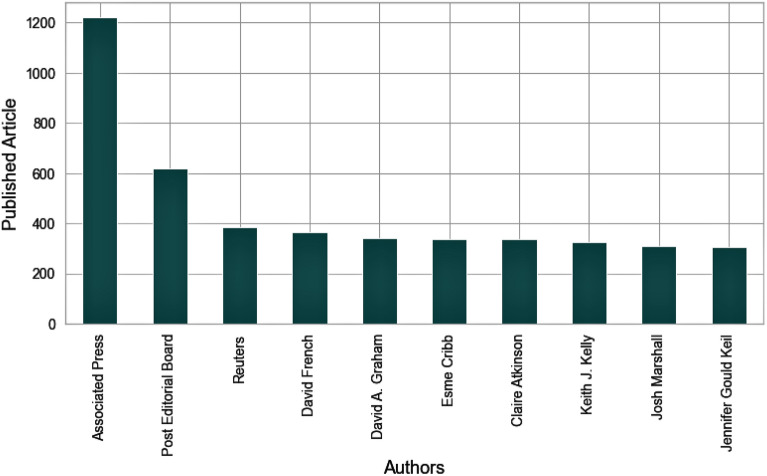
Top 10 authors in article2 dataset.

**Article2:** The article2 dataset contains 7 publications which consist of 50,000 articles from 4910 authors. In this dataset, New York Post and Atlantic are the top 2 publications with 17,493 and 7008 articles in the article2 dataset. The top 10 authors with the most publications are presented in Fig. 4. There is a 733 average number of words in the article2 dataset, where the longest article contains 29,790 words.

**Figure 5:**
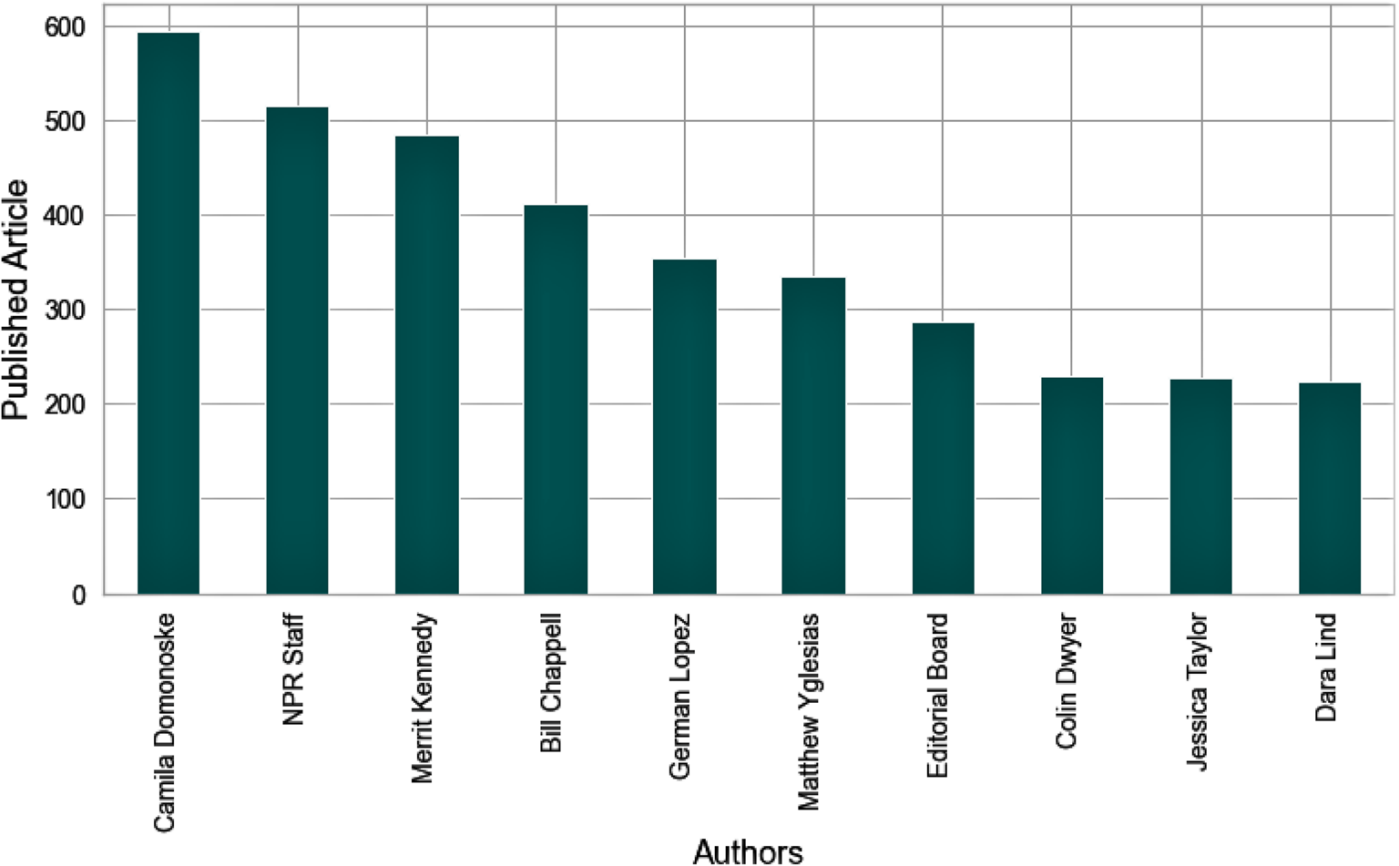
Top 10 authors in article3 dataset.

**Article3:** There are 5 publications in artcle3 dataset containing 50,000 articles from 7895 authors. The Non-Profit (NPR) and Washington Post are the top 2 publications with 11,992 and 11,114 articles. Figure 5 shows the top 10 authors that have the most publications in the article3 dataset. In the article3 dataset, the average number of words in an article is 734, and the longest article in the article3 dataset has 51,499 words.

## Proposed approach

**Figure 6:**
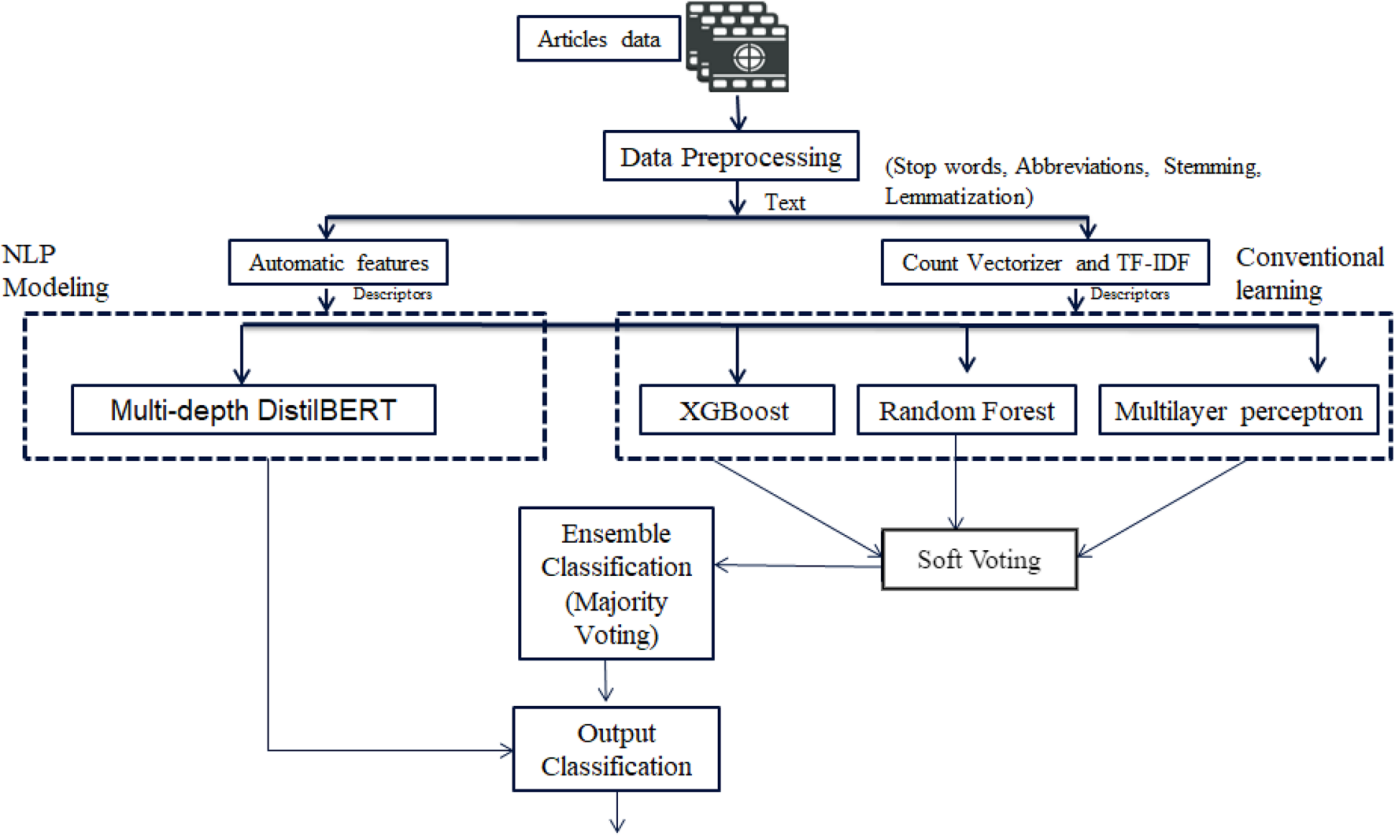
Graphical Representation of Proposed Approach for Authorship identification and classification.

The proposed approach depends on multiple phases. The first step is data preprocessing to handle imbalanced class data, overfitting data, handling missing values, and handling a large and noisy dataset; the second step is the selection of useful features using the supreme feature engineering technique to extract the most important features that help the model for classification and identification of actual authors. The last step is the model selection to identify the author of the actual text. The proposed ensemble method and multi-depth *DistilBERT* model performed well on the “All the news” dataset with higher accuracy than baseline approaches. Figure 6 depicts the proposed approach. The methods used in the proposed approach are explained below.

### Data pre-processing

The textual data sometimes may be noisier, and it requires appropriate data preparation for better classification purposes. The authorship analysis and identification are based upon the particular writing style of every author. We need to analyze the data in a way that does not change the actual meaning of the sentence and also does not change the author’s writing style. The various pre-processing steps were taken (identify missing values, check for duplicated values, and many more).

1. One way to analyze the “All the news” dataset is by calculating word frequency to know how frequently words appear in an article. It is a key component to understanding the relevancy of a given article and its actual author.
2. To understand the text’s context and convert the words to their meaningful base form, we use lemmatization. It is used with the nltk technique that converts words to their base form, for example, “played” to “play.”
3. Stop words are generally utilized in NLP to remove words that do not carry much helpful information. In the third step of pre-processing, we remove stop words to overcome the noise, such as (“is,” “a,” “the”). The removal of stop words does not affect the actual meaning of the sentence.
4. Due to word capitalization, sometimes it understands the same word as two different words. The model cannot differentiate between uppercase words and lower case words. To avoid this, we adjusted and converted all capital words to lower case words that do not change the meaning of the actual word.
5. Most authors used shortened forms and abbreviations of words in the text. We apply the contraction mapping technique to shorten the words or phrases by dropping or replacing a letter with an apostrophe. The contraction mapping is the process that drops the vowels from words. Contraction mapping is essential while working with textual data.
6. We use textblob because it provides noun phrase extraction, part-of-speech tagging, and sentiment analysis. The primary step of pre-processing is Part-of-speech (POS) tagging. It builds the parse trees used to construct “most named entities are nouns” (NERS) and extracts the relationship between words. It is also used for applying lemmatizers to return a word to its base form.

### Feature extraction

The goal of feature extraction is to extract the most important features from a dataset for better classification, and authorship identification^32–34^. This study uses a Count vectorizer and bigram TF-IDF techniques for feature extraction. First, we use the count vectorizer feature extraction technique, which counts the most frequent terms in the dataset and converts text into a vector based on frequently occurring terms or words (count).

The count vectorizer represents a word matrix. In this matrix, the columns are represented by unique words in the text, and the word count of the text represents the rows. For example, “The Queen is not ready to attend church as she is still recuperating from a heavy cold” the words ’is’ repeated twice and we have got this particular word count as 2 and 1 for the rest. This is the way we count words from a particular text. In this study, we essentially used the default parameters of the count vectorizer feature extraction approach, which involves removing stop words from the data, removing punctuation marks, and converting uppercase to lowercase characters. Furthermore, along with the count vectorizer, we use the TF-IDF feature extraction technique to extract the important features from textual data. We used TF-IDF for text analysis; it extracts weighted features for boosting the execution process^35^. The TF-IDF technique’s weighted features take a dot product of term-frequency and inverse document frequency. The frequency count of features in a particular text document is Term-Frequency (Tf). The TF-IDF parameters are max_df value is 0.5, the minimum df value is 2 and the ngrams=(1, 1), all other parameters remain default. The TF is defined in the Eq. (1). Here $count_{t,d}$ represents the total number of term frequency t in document d. The $totalcount_{d}$ is the count of several terms in document d. The IDF identified the increase in term t, which is more informative during the model training

1 $$\begin{aligned} {TF = \frac{count_{t,d}}{totalcount_{d}}} \end{aligned}$$

The idf estimates the increase of term t being more tremendous informative in the report for model training as defined in Eq. (2).

2 $$\begin{aligned} {idf = {i}/{df_{t}}} \end{aligned}$$

The *i* represents the total number of documents where $df_{t}$ presents the document that contains the term t. The IDF gets the low weight of term t when many documents contain the same common term t. Stop words with low idf value are the best example of this. TF-IDF is defined in Eq. (3).

3 $$\begin{aligned} {tf - idf = tf_{t,d} * \log (idf) } \end{aligned}$$

We use *DistilBERT* transformer-based model and trained it on large sizes of data. Furthermore, We use multi-depth *DistilBERT* transformer-based model. We fine-tune this pre-trained model. We reduce the size of the BERT model up to 40% by retaining its language understanding capabilities by 97%, but *DistilBERT* transformer-based model is 60% faster than BERT. The automated feature is a tensor array retrieved after tokenization, padding, and masking and then passed to the *DistilBERT* model for classification.

### Classification methods

The models used for authorship identification are described below. We present a description as well as the parameter setting of each model.

**Logistic regression:** It used the logit function and predicted the probability of the discrete classes. It is a supervised learning algorithm. It uses a logistic sigmoid function to predict the target class. It is a machine learning model used widely for classification purposes such as authorship analysis, cancer identification, and diabetes detection. We used different parameters of the Logistic regression algorithm; 0 verbose, 100 maxiter, lbfgs solver, 1.0 C, and 12 penalty.

**Multi-depth DistilBERT:** In this study, we implemented a pre-trained multi-depth *DistilBERT* transformation model. Based on the previously existing models like BERT, we carry out various modifications by reducing the number of layers and feeding the last layer token-type embeddings for each token. The results show that embedding embeddings from various layers provide higher representations and boost the model’s overall performance. The fine-tuned parameters of the proposed multi-depth *DistilBERT* model are as follows: epochs are set to 3, batch size to 80, learning rate to 5e–05, accumulation steps to 4, random seed to 42. For our “All the news” dataset, we employed the distilbert-base-uncased model. This model was applied to three distinct subsets of the “All the news” dataset. This model is uncased, which means it does not distinguish between *English* and *english*. We divided our data into train, test, and validation categories. Training and testing sessions are not included in the validation data. The amount of the validation data is unimportant because it does not affect the accuracy value. The training set is 70%, the testing set is 20%, and the validation set is 10%. For example, if we have 10 authors and 5000 articles, 3600 articles are used to train the *DistilBERT* model, 1000 articles are used to test the model, and 400 articles are used to validate the model. In each scenario of experiments, the dataset is divided into training, testing, and validation and fed to the *DistilBERT* model.

### Ensemble classifiers

Ensemble learning is a prevalent technique in the research domain among researchers^32, 36, 37^. This study used different machine learning algorithms for ensemble learning purposes^38, 39^. Ensemble learning is used to identify authors explicitly related to the article. We combine different ML classifiers and achieve better classification performance based on the voting mechanisms^40^. We used a majority voting mechanism in which every single classifier in an ensemble learning predicts a class label when we get a new variable or instance. The class with high classifier prediction or majority votes is assigned as the target label of that variable or instance. The ensemble learning achieves better performance than the conventional single ML model.

The proposed ensemble learning approach combines multiple classifiers’ predictions, and the final output depends upon the majority voting mechanism. We fine-tuned every single classifier to get a better result. The majority voting mechanism is based upon the Eq. (4).

4 $$\begin{aligned} {\overset{\sim }{y} = argmax(N_{c}(y_{t}^{1}),N_{c}(y_{t}^{2}),....,N_{c}(y_{t}^{n}))} \end{aligned}$$

The Eq. (4) ${N_{c}(y_{t})}$ presents the class that gets the most number of votes. We used XGBoost, Random Forest, and Multilayer Perceptron classifier for ensemble learning.

**Random forest:** Random Forest is a classification algorithm used for the ensemble learning method. It is an ML ensemble classifier used for multiple tasks classification regression. It works by building several decision trees that utilize as an ensemble, and the output target label depends upon the votes taken from those trees^41^. Random Forest decreases the over-fitting problem by making several decision trees. RF is also used to deal with complex data, unlike conventional ML classifiers. In the RF parameter setting, we set n_estimatores to 100, bootstrap to True, the criterion to Gini, min-samples-leaf to 1, min-samples-split to 2, and random-state to None.

**Extreme gradient boosting:** Carries efficiency and memory resources. It is based on multiple trees; due to this, it gained attention in recent years. It consists of many weak learners that are parallelly working because of this mechanism. XGboost is faster and gives more speed boost up. The ensemble algorithm uses Extreme Gradient Boosting to improve better classification performance. Extreme Gradient Boosting is a unique model that combines weak learning models into a stronger one. At each iteration, the residual error is optimized based on the previous predictor and optimized the loss function. We used L1 and L2 Regularizar to handle overfitting, which is defined as

5 $$\begin{aligned} J(\Theta )=L(\Theta )+\Omega (\Theta ). \end{aligned}$$

In the Eq. (5), $\Theta$ represents the trained parameters on the given data. *L* shows the training loss function, and to calculate model complexity $\omega$ regularization term is added. For Xgb, we set the parameters as follows: booster to gbtree eta to 0.3, min-child-weight to 1, max-depth to 6, and scale-pos-weight to 1.

**Multi-layer perceptron (MLP):** is mainly used for classification and prediction problems. To train on dataset it used function ${F(X): R^{n}\rightarrow {R^o} }$. The *o* is the total output dimensions, where *n* is the input dimensions. We used a feature set $X=x_{1},x_{2},x_{3}....x_{n}$ with the target variable *y*. Every node is called a neuron, and it has a nonlinear activation function used for the classification or regression process. The parameters of the MLP model are presented as follows: Activation to relu, solver to Adam, alpha to 0.0001, max-iter to 200, shuffle to True, and verbose to False.

### Proposed ensemble algorithm

Let *D* denoted the dataset containing instances $I={i_{1},i_{2}....i_{n}}$. *CP* represents the prediction confidence of each model. *CT* is the threshold to evaluate the *CP* of every single model. *LT* is the number of target classes to be predicted by each classifier. *NTL* denotes the total number of classes. Let *CI* be the total number of instances. The count of each class is incremented when a voting classifier votes for the target class, which *TIC* denotes. *I* denotes each attribute that is an input to the classifier, and it is appended in *CI*. After that, we evaluated the predicted confidence of *TIC* and *TL*. Every single classifier gives the vote to each observation. We set 80% as the threshold value to compare the confidence. The instance value must attain the threshold value of 80% or above to fall in a particular class. If it does not meet the essential criteria, a new instance is added until the requirement does not meet. Suppose one or more than one instance participates in the prediction result and achieves the same number of votes, then a random selection process is applied to select a random instance. If the *PCL* value is higher than the 80%, it considers the target class as a label of that corresponding instance. 
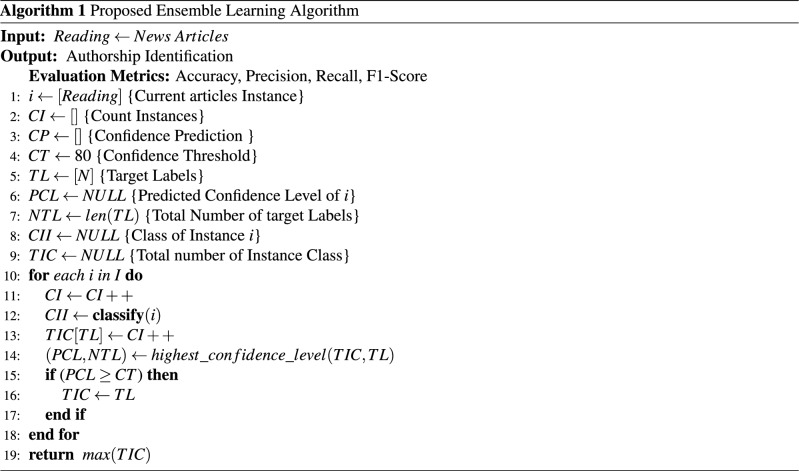


## Experimental analysis and results

The multi-depth DistilBERT and ensemble learning approach mainly classify and identify authors from the dataset. This study used three subsets of the “All the news” dataset, applied different approaches to all three subsets, and analyzed their performance using various evaluation measures. First, we describe different experiments resulting from applying different algorithms to each feature extraction. We also evaluate from experiments which features gave a high performance. We performed experiments using various machine learning models. Experiments are performed using multiple machine learning algorithms, i.e., Random Forest, XGboost, MLP, LR, proposed ensemble method, and multi-depth *DistilBERT* model on all the three subsets of the dataset. Once we have done experimentation, we compare the results with the state-of-the-art method^42^. We used accuracy, precision, recall, and f1-score as performance evaluation metrics. We use these metrics to check the model’s capabilities to produce the best classification results. The computing environment for the experimentation is presented in Table 1.

**Table 1:** Environment setup.

Parameters	Setting
COding framework	Jupyter notebook
OS	Windows 10 Home
CPU	Xeon Processor
GPU	NVIDIA GeForce GTX 1050
RAM	8GB
Programming Language	Python
Python Version	3.8

### Results

This study used three datasets (article1, article2, and article3), the subsets of the “All the news” dataset. We analyze the performance of different machine learning and transformer-based models on all three subsets of the “All the news” dataset. The ensemble learning approach and transformer-based model performed well on the “All the news” dataset. It classifies the dataset into its respective categories and identifies the authors of the actual text. The models are trained using 80 percent of the dataset, while the models are tested using 20 percent of the dataset. The Accuracy, Precision, Recall, and F1-score is the assessment measures used in the experiments to evaluate the model performance. We repeated the experiments several times to assess the better performance of the algorithms using various evaluation metrics utilized in this work.

#### Article1

For experimentation, we limited the scope of the dataset. We limited the scope into two steps; the top ten authors are selected in the first step. 500 news articles are selected from each author, so the total number of articles is 5000. 4000 articles are used to train the model, and 1000 articles are used to test the model using count vectorizer features and TF-IDF features. The output matrix of training count vectorizer features is 4000 $\times$ 53,396 with 675,039 stored elements in Compressed Sparse Row format, and TF-IDF training features sparse matrix is 4000 $\times$ 21,554 with 606,991 stored elements. The testing features using count vectorizer are 1000 $\times$ 53,396 with 157,806 stored elements in Compressed Sparse Row format, and with TF-IDF are 1000 $\times$ 21,554 matrices with 146,308 elements. In the next step, we selected the top 20 authors, and from each author, 100 articles were selected, so the total number of articles from 20 authors is 2000. 1600 $\times$ 38,359 is the sparse matrix with count vectorizer and 319,176 stored elements in Compressed Sparse Row format, same as for testing the sparse matrix of 400 $\times$ 38,359 with 74,073 stored elements in Compressed Sparse Row format. Similarly, the TF-IDF feature matrix for training is 1600 $\times$ 15,963 with 281,603 elements, while for testing, 72,251 elements are stored in a matrix of 400 $\times$ 15,963.

Table 2 shows the results of the first step in which the top 10 authors and a total of 5000 news articles are selected. Table 3 shows the results of the second step in which the top 20 authors and a total of 2000 news articles are selected. The ensemble model achieves the highest accuracy of 97% compared to others using Count vectorizer and separately TF-IDF features extraction techniques. We also measure other evaluation metrics like precision, recall, and f1-score. The precision, recall, and f1-score of the ensemble learning model using feature extraction techniques, count vectorizer, and TF-IDF are 97%, 97%, and 97%, which is also higher than other models. The ensemble model achieves the highest accuracy of 79% using the Count vectorizer compared to other models and the TF-IDF feature extraction technique. We also used other evaluation metrics like precision, recall, and f1-score. The precision, recall, and f1-score of the ensemble learning model using the count vectorizer technique are 81%, 79%, and 79%, which is also higher than the TF-IDF feature extraction technique and other models.

**Table 2:** All Feature Extraction and algorithm Results for Article1 Dataset with 10 Authors.

Algorithm	Features	Accuracy%	Recall%	Precision%	F1-score%
RF	Count vec	95	95	96	95
	TF-IDF	95	95	95	95
XGB	Count vec	96	96	96	96
	TF-IDF	96	95	95	95
MLP	Count vec	94	94	94	94
	TF-IDF	94	94	94	94
LR	Count vec	95	95	96	95
	TF-IDF	94	94	95	94
Ensemble	Count vec	97	97	97	97
	TF-IDF	97	97	97	97
*DistilBERT*	*DistilBERT*	89	89	90	89

**Table 3:** All Feature Extraction and algorithm Results for Article1 Dataset with 20 Authors.

Algorithm	Features	Accuracy%	Recall%	Precision%	F1-score%
RF	Count vec	73	73	75	72
	TF-IDF	73	73	75	72
XGB	Count vec	73	72	73	73
	TF-IDF	72	72	71	71
MLP	Count vec	74	74	78	75
	TF-IDF	72	72	72	71
LR	Count vec	76	76	76	76
	TF-IDF	70	70	70	69
Ensemble	Count vec	79	79	81	79
	TF-IDF	75	75	74	74
DistilBERT	*DistilBERT*	77	77	79	76

#### Article2

We divide the experimentation process into two stages to evaluate the proposed approach’s classification performance on the article2 dataset. In the first stage, the top 10 unique authors are selected, and each author contains more than 300 articles, so the total number of published articles is 3698 with training count vectorizer sparse matrix is 2958 $\times$ 49,921 and 572,093 stored elements and testing sparse matrix of 740 $\times$ 49,921 with 137,402 stored elements in it. The TF-IDF features used to train the model have 531,601 elements stored in 2956 $\times$ 23,153 sparse matrix, and the testing features have a sparse matrix of 740 $\times$ 23,153 with 127,788 stored elements. In the second stage of the experiments, we selected the top 20 authors, and from each author, 100 articles were selected, so the total number of articles from 20 authors is 2000 with training count vectorizer sparse matrix is 1600 $\times$ 43,022 and 456,964 stored elements and testing sparse matrix of 400 $\times$ 43,022 with 105,566 stored elements in it. Similarity used 1599 $\times$ 21,162 TF-IDF sparse matrix to train the model and 400 $\times$ 21,162 sparse matrices to test the models.

Table 4 depicts the results of the first stage in which the top 10 authors and a total of 3000 news articles are selected. The *DistilBERT* model achieves the highest accuracy of 94% compared to other feature extraction techniques and conventional ML and ensemble learning models. We also measured precision, recall, and f1-score score. The precision, recall, and f1-score of the *DistilBERT* model are 95%, 94%, and 94%, which is also higher than other models. The results of the second stage are presented in Table 5 in which the top 20 authors and a total of 2000 news articles are selected. We achieve the highest accuracy of 90% using the *DistilBERT* model compared to other models and feature extraction techniques. The Dilbert model gives the highest score of 95%, 90%, and 90% in the form of precision, recall, and f1-score compared to other algorithms.

**Table 4:** All Feature Extraction and algorithm Results for Article2 Dataset with 10 Authors.

Algorithm	Features	Accuracy%	Recall%	Precision%	F1-score%
RF	Count vec	85	86	86	86
	TF-IDF	80	80	81	79
XGB	Count vec	87	88	88	88
	TF-IDF	83	83	84	83
MLP	Count vec	86	86	87	86
	TF-IDF	85	85	85	85
LR	Count vec	86	86	86	86
	TF-IDF	82	82	83	82
Ensemble	Count vec	89	89	90	90
	TF-IDF	89	89	89	89
*DistilBERT*	*DistilBERT*	94	94	95	94

**Table 5:** All Feature Extraction and algorithm Results for Article2 Dataset with 20 Authors.

Algorithm	Features	Accuracy%	Recall%	Precision%	F1-score%
RF	Count vec	78	78	79	77
	TF-IDF	76	76	77	75
XGB	Count vec	80	80	80	80
	TF-IDF	76	76	78	75
MLP	Count vec	85	85	85	84
	TF-IDF	80	81	83	81
LR	Count vec	86	86	86	86
	TF-IDF	79	79	81	79
Ensemble	Count vec	87	87	87	86
	TF-IDF	82	82	84	83
*DistilBERT*	*DistilBERT*	90	90	95	90

#### Article3

In the Article3 dataset, we performed experiments in two parts. First of all, the top 10 authors are selected, and from each author, more than 200 articles are selected, which results in a total of 3552 published news articles and a training count vectorizer sparse matrix of 2841 $\times$ 61,328 with 799,298 stored elements and testing sparse matrix of 711 $\times$ 61,328 with 201,120 stored elements. However, we also used TF-IDF features, which have a training features matrix is 2841 $\times$ 26,278 with 743,984 elements, and the testing features matrix is 711 $\times$ 26,278 with 188,372 elements. Secondly, we selected 20 authors, and every single author contains 100 articles with a total of 2000 published news articles with training count vectorizer sparse matrix of 1600 $\times$ 46,777 with 521,540 stored elements and testing sparse matrix of 400 $\times$ 46,777 with 116,929 stored elements. The TF-IDF training feature matrix is 1600 $\times$ 20,838, and the testing feature matrix is 400 $\times$ 20,838.

Table 6 shows the results of the top ten authors. We used a count vectorizer with an ensemble approach and got the highest accuracy of 85% than TF-IDF and other conventional machine learning and transformer-based model. We also used precision, recall, and f1-score evaluation metrics. Ensemble model achieves 85% precision score, 85% recall, and 84% f1-score. Table 7 represents the second part of the experimentation process in which we selected 20 authors and 2000 news articles. We achieve the highest accuracy of 74% using the ensemble learning model and count vectorizer features. It also achieves the highest precision, recall, and f1-score. The highest precision, recall, and f1-score are 76%, 74%, and 74%.

**Table 6:** All Feature Extraction and algorithm Results for Article3 Dataset with 10 Authors.

Algorithm	Features	Accuracy%	Recall%	Precision%	F1-score%
RF	Count vec	71	71	75	69
	TF-IDF	71	71	72	69
XGB	Count vec	81	81	81	81
	TF-IDF	80	80	80	80
MLP	Count vec	79	79	79	79
	TF-IDF	75	75	77	74
LR	Count vec	80	80	81	81
	TF-IDF	70	70	74	68
Ensemble	Count vec	85	85	85	84
	TF-IDF	83	83	84	82
*DistilBERT*	*DistilBERT*	70	69	72	69

**Table 7:** All Feature Extraction and algorithm Results for Article3 Dataset with 20 Authors.

Algorithm	Features	Accuracy%	Recall%	Precision%	F1-score%
RF	Count vec	64	64	66	62
	TF-IDF	64	64	69	63
XGB	Count vec	68	68	68	68
	TF-IDF	66	66	67	66
MLP	Count vec	73	73	75	73
	TF-IDF	71	70	71	69
LR	Count vec	70	70	71	70
	TF-IDF	66	66	69	65
Ensemble	Count vec	74	74	76	74
	TF-IDF	73	73	75	73
*DistilBERT*	*DistilBERT*	65	65	80	66

### Comparative analysis with baseline approach

To analyze the classification performance of the proposed ensemble learning approach and transformer-based multi-depth *DistilBERT* model, we compare the result with the state-of-art study^42^. The experimental settings of the proposed approach and baseline approach resembled each other. The comparison of the proposed approach and baseline approach is presented in Table 8 in which the baseline approach limited the scope of the “All the news” dataset. The “All the news” dataset has three subsets (article1, article2, and article3), but the authors performed experiments using only the article1 dataset. They limited the scope and selected only the top 10 authors from the article1 dataset, and also they selected 500 news articles from every single author, which became a total of 5000 news articles.

**Table 8:** Performance comparison of proposed approach and baseline approach with 10 authors.

Algorithm	Features	Accuracy%	Recall%	Precision%	F1-score%
**Baseline approach**					
RF	BOW	92.66	95.2	92.66	93.87
	LSA	73.2	76.13	73.2	74.01
SVM	BOW	92	95.6	92	92.43
	LSA	78.66	83.86	78.66	79.27
LR	BOW	93.86	96.13	93.86	94.10
	LSA	82.53	84.62	82.53	82.70
BERT	BERT	86.56	85.19	88	86
**Proposed approach**					
Ensemble	Count vec	97	97	97	97
	TF-IDF	97	97	97	97
*DistilBERT*	*DistilBERT*	89	89	90	89
Gain		3.14	0.87	3.14	2.90

**Figure 7:**
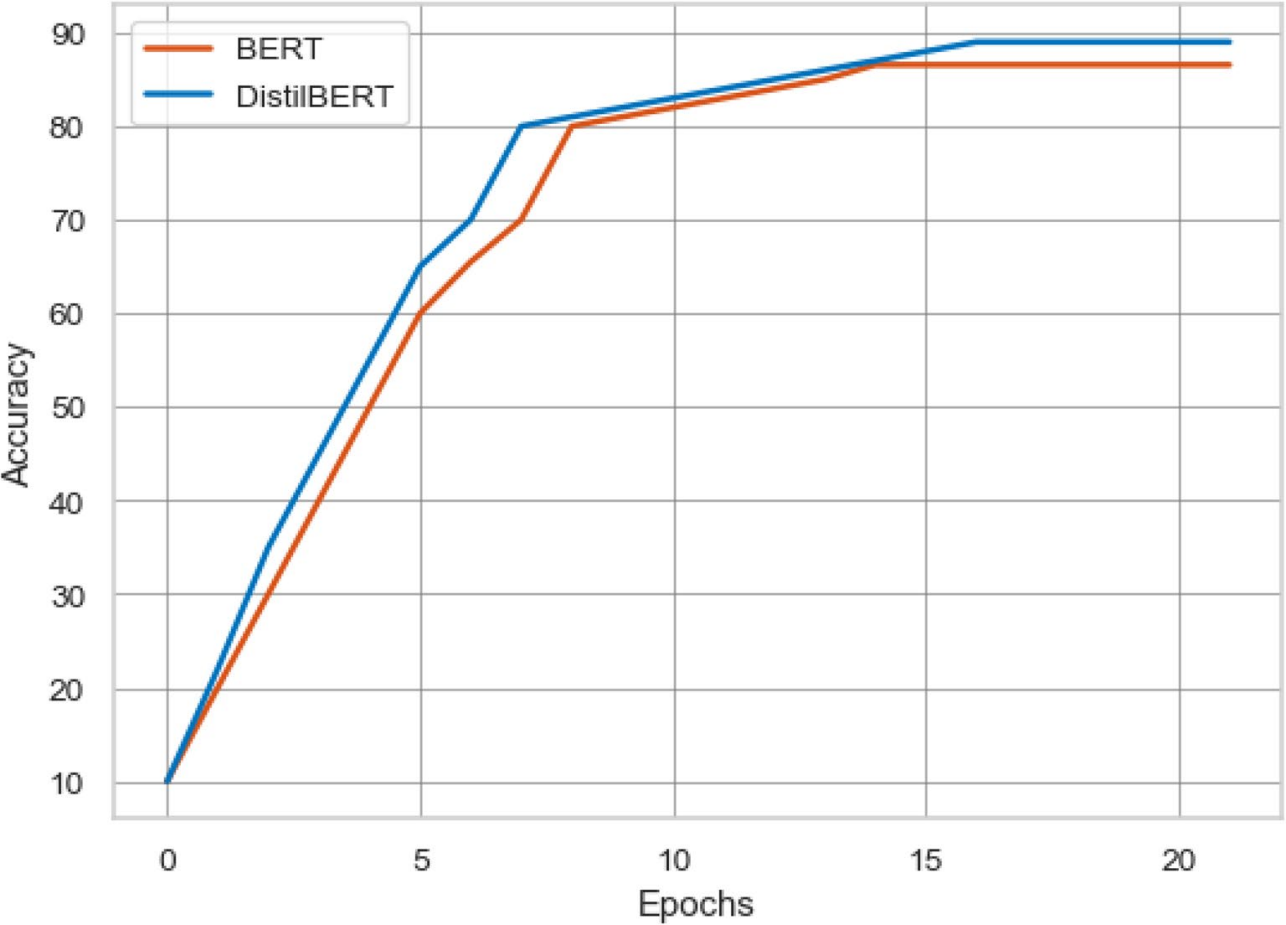
Accuracy comparison of transformer-based models using top 10 authors.

**Table 9:** Performance comparison of proposed approach and baseline approach with 20 authors.

Algorithm	Features	Accuracy%	Recall%	Precision%	F1-score%
**Baseline approach**					
RF	BOW	72	72	73.3	72
	LSA	58	58	56.55	58
SVM	BOW	70	70	70.87	70
	LSA	58.33	58.33	59.85	58
LR	BOW	74	74	74.20	74
	LSA	65	65	64.63	65
BERT	BERT	70.33	66.56	70.40	67
**Proposed approach**					
Ensemble	Count vec	79	79	81	79
	TF-IDF	75	75	74	74
*DistilBERT*	*DistilBERT*	77	77	79	76
Gain		5.25	5.00	6.80	5.00

Compared to the baseline approach, our experimental settings are the same, but we performed experiments using all three subsets and performed very well compared to baseline approaches. The baseline approach used RF, SVM, LR, and BERT models for author identification, and they achieved the highest accuracy of 93.86% using BOW features and the LR model. Compared to baseline results, we achieved the highest accuracy of 97% using the proposed ensemble model and both count vectorizer and TF-IDF features with an accuracy gain of 3.14%. We also achieved the highest precision, recall, and f1-score of 97%, 97%, and 97% compared with the baseline results. The gain in terms of precision, recall, and f1-score is 3.14%, 0.87%, and 2.90%.

**Figure 8:**
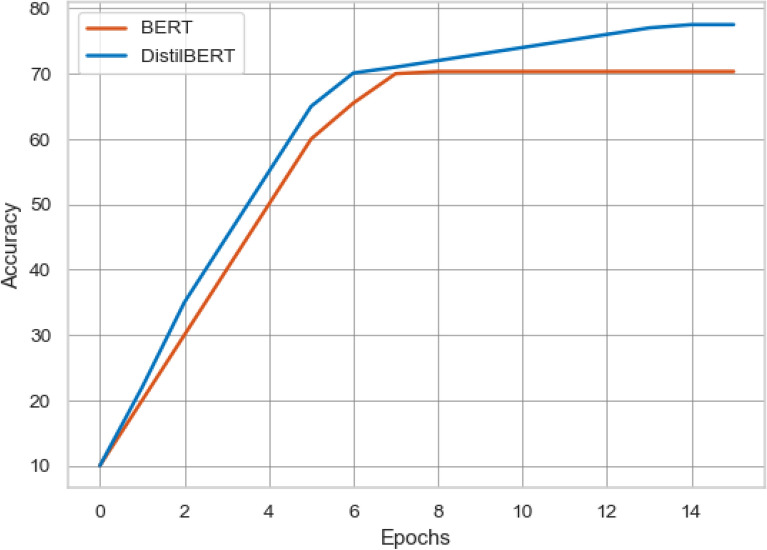
Accuracy comparison of transformer-based models using top 20 authors.

Figure 7 shows the comparison of transformer-based models of the proposed approach and baseline approach. The proposed approach based on NLP outperforms the baseline approach with an accuracy gain of 2.44%. In the second step of the experimentation, the baseline approach selected 20 unique authors and 2000 news articles from the article1 dataset for authorship identification. The result comparison of the proposed approach and baseline approach is presented in Table 9. The baseline approach gets the highest accuracy of 74% using the LR model and BOW features. Compared to the baseline highest results, our proposed ensemble model with count vectorizer features achieves the highest accuracy of 79% with the accuracy gain of 5.25%. The proposed ensemble model outperforms the baseline LR model in terms of precision, recall, and f1-score with 81%, 79%, 79% and with the gain of 6.80%, 5.00%, and 5.00%. Figure 8 shows the comparison of the baseline BERT model with the proposed multi-depth *DistilBERT* model. It is shown that the proposed *DistilBERT* model outperforms the baseline BERT model with an accuracy gain of 7.17%.

## Conclusion

Authorship identification refers to maintaining intellectual property rights, saving articles from theft, and referring each article to its specific author. It enables the establishments or institutes to provide author identification credit. The dataset used in this work consists of news articles named “All the news” dataset, which is available on kaggle. It needs some modifications before being fed to ML algorithms, such as handling missing values, removing duplication, removing stop words, and adjusting capitalization. Furthermore, the count vectorizer and bi-gram TF-IDF were used for specific feature extraction, and then compared the findings of both feature extraction techniques. This study analyzes various types of algorithms, Random Forest, Extreme Gradient Boosting, Multi-layer perceptron, logistic regression, ensemble learning, and Distil-BERT. The specific features are passed to models for authorship identification. We limited the scope of the dataset. The top 10 authors are selected in the first scope, and 20 unique authors are selected in the second. The proposed ensemble learning and transformer-based MultiDepth approach gave higher accuracy, precision, recall, and f1-score when compared with a similar state-of-the-art study. This achievement can seriously help in applying authorship’s analysis in real-life applications.

## Limitation and future work

This work can be extended to deep learning algorithms, and we plan to extend the scope to more than 20 authors and 10,000 articles to make a broad comparison between deep learning and machine learning algorithms.

This study also has a few limitations; the preceding work on this dataset is limited to only one research work^42^, so the dataset is new. There is a dataset used in the study^43^, but this is not the same dataset. The *DistilBERT* and ensemble learning algorithms are relatively new. There is no current work related to this approach for authorship identification by applying the *DistilBERT* and ensemble learning approach. We performed experiments using a *jupyter notebook*, so the computational complexity of our approach is very high. We also intend to reduce the computational cost of this approach in the future.

## Data Availability

The datasets analyzed during the current study are available in the Kaggle repository, [https://www.kaggle.com/snapcrack/all-the-news].
